# Combining information from surveys of several species to estimate the probability of freedom from *Echinococcus multilocularis *in Sweden, Finland and mainland Norway

**DOI:** 10.1186/1751-0147-53-9

**Published:** 2011-02-11

**Authors:** Helene Wahlström, Marja Isomursu, Gunilla Hallgren, Dan Christensson, Maria Cedersmyg, Anders Wallensten, Marika Hjertqvist, Rebecca K Davidson, Henrik Uhlhorn, Petter Hopp

**Affiliations:** 1 National Veterinary Institute, 752 89 Uppsala, Sweden; 2 Finnish Food Safety Authority Evira, Fish and Wildlife Health Research Unit, PL 517, 90101 Oulu, Finland; 3 Swedish Board of Agriculture, 551 82 Jönköping, Sweden; 4 Swedish Institute for Communicable Infectious Disease Control, 171 82 Stockholm, Sweden; 5 Norwegian Veterinary Institute, 0106 Oslo, Norway

## Abstract

**Background:**

The fox tapeworm *Echinococcus multilocularis *has foxes and other canids as definitive host and rodents as intermediate hosts. However, most mammals can be accidental intermediate hosts and the larval stage may cause serious disease in humans. The parasite has never been detected in Sweden, Finland and mainland Norway. All three countries require currently an anthelminthic treatment for dogs and cats prior to entry in order to prevent introduction of the parasite. Documentation of freedom from *E. multilocularis *is necessary for justification of the present import requirements.

**Methods:**

The probability that Sweden, Finland and mainland Norway were free from *E. multilocularis *and the sensitivity of the surveillance systems were estimated using scenario trees. Surveillance data from five animal species were included in the study: red fox (*Vulpes vulpes*), raccoon dog (*Nyctereutes procyonoides*), domestic pig, wild boar (*Sus scrofa*) and voles and lemmings (Arvicolinae).

**Results:**

The cumulative probability of freedom from EM in December 2009 was high in all three countries, 0.98 (95% CI 0.96-0.99) in Finland and 0.99 (0.97-0.995) in Sweden and 0.98 (0.95-0.99) in Norway.

**Conclusions:**

Results from the model confirm that there is a high probability that in 2009 the countries were free from *E. multilocularis*. The sensitivity analyses showed that the choice of the design prevalences in different infected populations was influential. Therefore more knowledge on expected prevalences for *E. multilocularis *in infected populations of different species is desirable to reduce residual uncertainty of the results.

## Background

**Figure 1:**
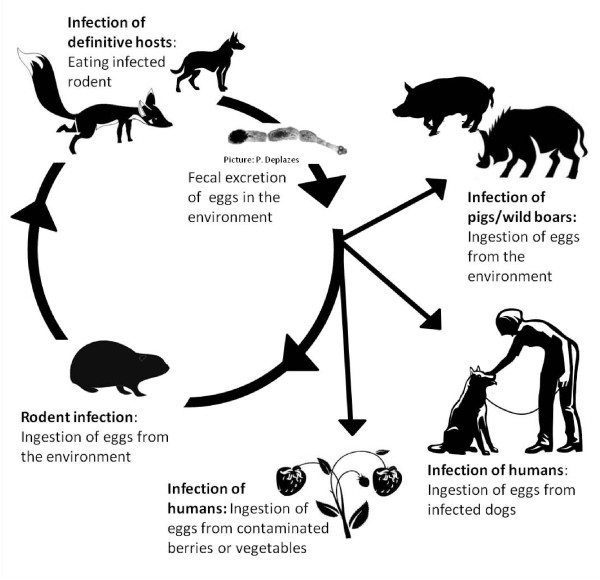
**Life cycle of *Echinococcus multilocularis***.

The fox tape worm *Echinococcus multilocularis *(EM) is a parasite of public health significance. The life cycle involves foxes and other canids as definitive hosts and rodents as intermediate hosts [1] although many other mammals species can be aberrant intermediate hosts (Figure 1). Humans become infected via the oral route, probably via contaminated hands after handling infected canids, contaminated plants or soil or through eating contaminated berries [1,2]. Human infection with EM can result in alveolar echinococcosis, a serious disease. If untreated the mortality exceeds 90% within 10 years, if treated the survival rate after five years increased to 88% [3].

EM is endemic in large parts of Europe [1] and the parasite is increasingly reported from countries near Sweden, Finland and Norway [4-7]. There is evidence that the parasite may be emerging in Europe [3,8-11]. EM is notifiable in humans and animals and has never been found in Sweden, Finland and mainland Norway. This favourable situation is probably largely attributed to the fact that this area is geographically isolated from countries where EM has been detected in combination with stringent import regulations including a requirement for anthelminthic treatment of companion animals (*i.e*. cats, dogs and ferrets). Furthermore EM has not been reported from adjacent areas of Russian Karelia, and according to Henttonen et al. [12], in all probability not on the Kola Peninsula. In Sweden, Finland and Norway, the climate is favourable for EM and susceptible hosts occur [13], hence it is possible that EM could be established if accidentally introduced. Once established in an area it is considered impossible to eradicate EM because of the sylvatic life cycle [14].

The present EU regulation allows Sweden, Finland, UK, Ireland and Malta to maintain national rules for the entry of companion animals over a transitional period to protect them from imported EM infections. In addition Norway (mainland) considers itself free from EM and has separate import regulations for pets from countries other than ones mentioned above. However, these special requirements may be costly and laborious for the pet owner and could be considered disproportionate. If a country wants to maintain stricter national import regulations for dogs and cats than EU generally, it should be able to plausibly demonstrate its freedom from EM. The aim of this study was to assess the EM status of Sweden, Finland and mainland Norway using past surveillance data. The study shows that there is a high probability that the three countries were free from EM in 2009.

## Methods

### Design of the study

**Figure 2:**
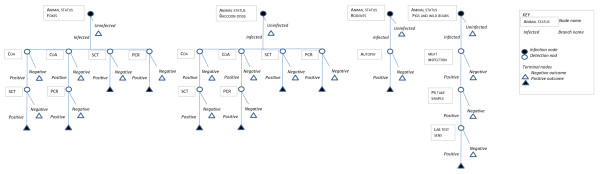
**Scenario trees describing surveillance systems for for *Echinococcus multilocularis *in Sweden, Finland and mainland Norway**. The number of animals, number of species and types of tests included in the surveillance differ between countries.

The probability that Sweden, Finland and mainland Norway are free of EM and the sensitivity of the surveillance systems for EM, *i.e*. the probability of case detection, were estimated using the method described by Martin et al. [15]. By use of modelling, the method allows combining results from several independent components of a complex surveillance system into a single measure; *i.e*. the sensitivity of the combined surveillance activities. The model can be graphically presented by scenario trees (Figure 2) that contain infection and detection nodes, and illustrate all possible pathways from the starting point (the population is infected) to the outcome (negative or positive test results) [15]. The model is based on two key assumptions: All results of the surveillance system are negative, *i.e*. disease is not detected, and the specificity of the surveillance system is 100%, *i.e*. each surveillance system component (*SSC*) (Table 1) is defined to include any necessary follow-up testing of potentially false positive results [15]. In the present study, the method was extended to combine information from surveillance systems in up to five different populations. A design prevalence was specified for each population surveyed (see further explanation below). Given the defined design prevalences (P*) the probability of freedom by country was calculated. The study period was from 1 January 2000 to 31 December 2009 and the surveillance for each year was modelled separately.

**Table 1:** Notations used in the model to quantify the probability of freedom from *Echinococcus multilocularis *in Sweden, Finland and mainland Norway

Notation	Explanation
P*_Sp_	Design prevalence at the animal level for species *Sp*.
*s*	Sample of animals of the same species tested with the same test during the same year.
*Sa_s_Se_Sp, t, y_*	Sample sensitivity: The probability of detection of EM in sample *s *of animals tested of species *Sp *with test *t *in year *y*.
*PIntro*	The annual probability of introduction and establishment of the infection in the country
*PostPFree*	The posterior probability of freedom from infection in the country
*PriorPInf*	Prior probability of infection in the country
*Sp*	Species: Species of animals (red foxes, raccoon dogs, domestic pigs, wild boars) or animal population (voles) included in the surveillance
*SSC*	Surveillance system component, the surveillance performed in one species
*SSCSe_Sp, y_*	The surveillance system component sensitivity for one species *Sp *for one year *y*
*SSSe _y_*	The surveillance system sensitivity: The combined sensitivity for all *SSC *for year *y*
*Se_t_*	The sensitivity of an individual test

### Input values

#### Number of animals examined

Five different animal-taxons, hereafter designated species, were included in the surveillance of EM: red fox (*Vulpes vulpes*), raccoon dog (*Nyctereutes procyonoides*), domestic pig, wild boar (*Sus scrofa*) and voles and lemmings (Arvicolinae, several species). Only pigs having access to pasture were considered to be exposed to EM and hence included in the study. The number of animals examined per year in each country is detailed in Table 2. A detailed description of the surveillance activities in each country is provided in the additional file 1: EM_DDF_Annex_datasources_2011_01_25.pdf

**Table 2:** The number of animals investigated for *Echinococcus multilocularis *in Sweden, Finland and mainland Norway

Sweden							
	**Foxes**		**Raccoon dogs**		**Pigs**	**Wild boars**	**Rodents**

**Year**	**CoA and SCT**	**SCT**	**SCT**		**Meat inspection**	**Meat inspection**	**Autopsy**

2000	0	11	0		8966	5310	0
2001	310	32	0		9428	10137	0
2002	313	0	0		9501	10331	0
2003	400	0	0		8639	16800	0
2004	400	0	0		8833	18344	0
2005	200	0	0		9893	22206	1000
2006	302	100	0		9434	23172	1000
2007	245	0	0		9369	22206	1000
2008	200	44	21		7804	31572	0
2009	305	0	28		6142	47310	0
Sum	2675	287	49		88009	207388	3000

**Finland**							

	**Foxes**		**Raccoon dogs**		**Pigs**	**Wild boars**	**Rodents**

**Year**	**CoA and SCT**	**CoA and egg PCR**	**CoA and SCT**	**CoA and egg PCR**	**Meat inspection**	**Meat inspection**	**Autopsy**

2000	9	0	0		4500	0	2000
2001	13	0	2		4000	1109	2000
2002	116	0	3		3500	1221	3000
2003	164	0	98		2500	788	650
2004	348	0	239		2000	1006	1850
2005	281	0	219		2500	486	3000
2006	209	0	193		2000	638	2100
2007	264	0	227		1700	373	2200
2008	0	411	0	148	1800	138	2100
2009	0	184	0	177	2000	286	800
Sum	1404	595	981	325	26500	6045	19700

**Norway**							

	**Foxes**		**Raccoon dogs**		**Pigs**	**Wild boars**	**Rodents**

**Year**	**CoA and egg PCR**	**Egg PCR**	**Egg PCR**		**Meat inspection**	**Meat inspection**	**Autopsy**

2000	0	0	0		825	0	0
2001	0	0	0		825	0	0
2002	85	0	0		825	0	0
2003	119	0	0		825	0	0
2004	104	1	0		1236	0	0
2005	5	0	0		1008	0	0
2006	0	31	0		1167	0	0
2007	0	539	1		1326	0	0
2008	0	455	0		745	0	0
2009	0	280	0		1238	0	0
Sum	313	1306	0		10020	0	0

The study period includes surveillance of the five different species in Sweden, Finland and mainland Norway from January 2000 to December 2009. The annual number of investigated animals is given per test and per species (CoA = coproantigen Elisa, SCT = sedimentation and counting technique, egg PCR = taeniid egg isolation and multiplex polymerase chain reaction).

#### Design prevalence

The design prevalence P* is the probability that an animal is infected given that the infection is present in the country. For each species, a separate design prevalence was specified (Table 3) based on prevalence estimates previously published. For foxes and raccoon dogs a design prevalence of 1% was used in agreement with the suggestions for harmonized monitoring of EM within the European Union [16].

**Table 3:** Input values used in the model to quantify the probability of freedom from *Echinococcus multilocularis*

Variables	Input values used in the model
Initial prior probability of freedom	0.5

*Design prevalences*	
Foxes	1%
Raccoon dogs	1%
Pigs with access to pasture	0.01%
Wild boars	0.02%
Rodents that are intermediate hosts for *E. multilocularis*	0.24%

*Test sensitivities*	
Coproantigen ELISA	Pert(0.4, 0.84, 0.93)
Sedimentation and counting technique	Pert(0.9, 0.98, 0.99)
PCR (Norway)	Pert (0.29, 0.5, 0.72)
PCR (Finland)	0.35 × Pert (0.29, 0.5, 0.72)
Dissection rodents (investigations in Finland)	Pert(0.8, 0.9, 0.99)
Dissection rodents (investigations in Sweden)	Pert(0.08, 0.09, 0.099)
Meat inspection of pigs	
Probability of detecting lesions at slaughter	Pert(0.01, 0.1, 0.2)
Probability of submitting a sample to laboratory	Pert(0.1, 0.2, 0.3)
Probability of diagnosing *E. multilocularis *in laboratory	Pert(0.1, 0.4, 0.5)
Meat inspection of wild boars	0.5 × the overall sensitivity of meat inspection of pigs

*Probability of introduction and establishment*	
Probability of introduction by dogs to Sweden	Pert(0.13, 0.45, 0.64)
Probability of introduction by dogs to Norway	0.5 × Pert(0.13, 0.45, 0.64)
Probability of introduction by dogs to Finland	0.75 × Pert(0.13, 0.45, 0.64)
Probability of introduction by wildlife to Finland	0.5 × 0.75 × Pert(0.13, 0.45, 0.64)
Probability of an infected dog excreting eggs	Pert(0.42, 0.6, 1)
Probability of an infected dog excreting eggs in a suitable environment	Pert (0.3, 0.5, 0.7)

For pigs the design prevalence was based on results from surveys for EM performed by inspection of pig livers at the slaughter house. In Hokkaido, between 1983 and 2007, approximately 0.1% of slaughtered pigs were reported to have livers with lesions due to EM [14,17,18]. In Lithuania, lesions due to EM were found in 0.5% of pigs (*n *= 612) from small family farms [19] and in Switzerland, livers from 10% of fattening pigs (*n *= 90) raised outdoors, originating from six farms with a high proportion of condemned livers, had EM lesions [20]. As these estimates originate from (high) endemic areas it was expected that the prevalence at the country level would be lower. Based on expert opinion, it was decided to use 10% of the lowest estimate, *i.e*. 0.01% as the design prevalence for the whole country.

Reports of EM found in wild boars [21,22] were not considered sufficient for estimation of the design prevalence. However, wild boars are expected to be more exposed to EM than domestic pigs, hence it was decided to use twice the design prevalence of pigs, *i.e*. 0.02%.

The prevalence estimates of EM in voles are reported to vary between as well as within species [23,24]. The common vole (*Microtus arvalis*) and water vole (*Arvicola amphibious *previously called *A. terrestris*) are considered to be the most important intermediate hosts in Central Europe [23-25]. Water vole is common in Nordic countries, but common vole does not occur in Sweden and Norway and has a very limited distribution in Finland. However, other *Microtus *species do occur in the area. Most of the rodents of this study (70-90%) were bank voles (*Myodes glareolus*), and the prevalence was set to fit that species. The reported prevalence estimates for this species varied from 2.4% to 10.3% [26-28]. In accordance with the reasoning for pigs, the design prevalence was set to 10% of the lowest estimate, *i.e*. 0.24%.

#### Test sensitivity

The sedimentation and counting technique (SCT) is considered the reference test for EM in definitive hosts. The sensitivity has been estimated to be 98% to 100% [24,29]. However the lower bound for the sensitivity (98%) as estimated by Eckert [30] was considered to be too high for a country where EM has never been diagnosed and therefore the personnel being less experienced (personal communication, Dan Christensson). In the present study, the sensitivity of SCT was therefore described with a Pert distribution with the parameters (0.9, 0.98, 1) [31].

The coproantigen ELISA (CoA) was estimated to have a sensitivity of 83.6% when investigating 87 wild foxes of which 55 were found positive in the SCT test [32]. In foxes with a detected parasite burden of > 21 worms the sensitivity of CoA reached 93.3%, but in foxes with ≤ 20 worms it was only 40% [32]. The sensitivity was described with a Pert distribution with parameters (0.40, 0.84, 0.93). The same estimates for sensitivity for CoA were used for foxes and raccoon dogs as the excretion of coproantigen is not expected to vary significantly between these species [33].

The overall diagnostic sensitivity of the modified taeniid egg isolation from faeces [34,35] and multiplex PCR [36,37] used in Norway was described by Pert distribution with the parameters 0.29, 0.5 and 0.72 [38]. In Finland, a modified taeniid egg isolation (McMaster with sucrose, specific gravity 1.25) was used with a sensitivity that was assumed to be 35% of the method used in Norway [19].

Meat inspectors in Sweden, Norway and Finland are not expected to be familiar with the white nodular lesions in the pig liver caused by EM. The pathological characteristics in pigs, an aberrant intermediate hosts, differ from rodents, the natural intermediate host. Most of the detected lesions have been described as small and calcified and may look similar to non-essential lesions such as "white spots" caused by passage of ascarid larvae [17]. Therefore the probability that EM lesions would be detected during meat inspection was estimated to be approximately 0.1 and was described by a Pert distribution with parameters 0.01, 0.1 and 0.2. The probability of taking a sample for further examination varies among the countries. In Norway, laboratory examination of samples is free only when a notifiable disease is suspected and the probability that a sample would be taken was considered to be very low and was described by a Pert distribution with parameters 0.1, 0.2 0.3, based on estimates from meat inspectors. In Sweden and Finland, the probability of sampling was expected to be higher as all samples can be submitted for further examination without any costs. However, as the probability is difficult to estimate, a conservative approach was chosen and the estimate for Norway was used for all three countries (Table 3).

Identification of EM lesions in pigs and wild boars by histological examination can be very difficult as older lesions very often are calcified and only fragments of the laminated layer of the parasite can be found. It can be expected that such lesions will not be identified by pathologists unfamiliar with EM. If a PCR is done on all putative lesions, the sensitivity of laboratory examinations is estimated to be a minimum of 80%, most likely 90% and a maximum of 95% (personal communication, Peter Deplazes). However, as EM has never been diagnosed in any of the three countries, it cannot be expected that all potentially suspect lesions will be submitted for further examination by PCR. The probability of diagnosing EM, if an EM lesion was sent to the lab, was estimated to be rather low and was described by Pert distribution with parameters (0.1, 0.4, 0.5) (Table 3). In wild boars, meat inspection is usually performed by laymen and the overall sensitivity of meat inspection was considered to be lower, we assumed it to be 50% of the sensitivity in domestic pigs.

In Finland, voles were dissected as part of regular long-term surveillance of small rodent populations by the Finnish Forest Research Institute. Voles were dissected by experienced biologists paying special attention to liver lesions. Thus, parasitic cysts were reliably investigated and identified at the species or genus level by morphology, sometimes also genetically for *Taenia *phylogenetics [39]. Consequently, the sensitivity of dissections, *i.e*. the probability of detecting a liver lesion due to EM, is estimated to be high and was described by Pert distribution with parameters (0.8, 0.9, 0.99) (personal communication, Heikki Henttonen) (Table 3). As the dissections of rodents in Sweden were performed by laymen, the sensitivity was estimated to be 10% compared to the estimate in Finland (Table 3).

#### Probability of introduction

In Sweden and Norway, dogs that are introduced from countries where EM is endemic and that do not comply with import requirements, are considered to be the most important pathway for introduction of EM. In Finland, the risk of introduction by wildlife is also considered important as EM is now present in neighbouring Estonia [40]. The annual risk of introducing at least one infected dog to Sweden has, depending on the degree of compliance with the import requirements, been estimated to be 0.64, 0.45 and 0.13 assuming 90%, 95% or 99.9% compliance, respectively [41]. The degree of compliance is unknown. In the UK it is estimated to be approximately 95% to 96% (personal communication, Tonima Saha). A risk of introduction of minimum of 0.13, most likely 0.45 and a maximum of 0.64, based on a 99%, 95% and 90% compliance was therefore used in this study.

The probability of establishment was considered to be dependent on the probability of infected dogs excreting eggs and the probability of excreted eggs starting an endemic cycle. Of the total infection period in dogs of approximately 120 days, the prepatent period constitutes approximately 28 days and the effective patent period approximately 43 days (95% CI 21.9-93.1) [33,42]. Therefore, it was assumed that dogs imported after 71 (28+43) days post infection would excrete very few eggs and were assumed to not initiate an endemic cycle. Consequently, approximately 60% (71/120) (95% CI 42% (49.9/120) -100% (121/120) of imported infected dogs would excrete sufficient eggs for initiating an endemic cycle. Furthermore, it was expected that the risk of initiating an endemic cycle would differ depending on the presence and number of suitable hosts. As no data were available, it was estimated that 50% (minimum 30% and maximum 70%) of infected dogs would excrete eggs in areas suitable to initiate an endemic cycle. Hence, the risk of introduction and establishment by dogs was described as a conditional probability parameterised with:

Pert(0.13, 0.45, 0.64) × Pert(0.42, 0.6, 1) × Pert(0.3, 0.5, 0.7) Where Pert(0.13, 0.45, 0.64) is the risk of introduction, Pert(0.42, 0.6, 1) is the probability that an introduced infected dog would excrete eggs and Pert(0.3, 0.5, 0.7) is the probability that an endemic cycle is initiated given introduction of an infected dog excreting eggs (Table 3). The estimated total number of dogs in Norway and Finland are approximately 50% and 75%, respectively, of the Swedish populations and the number of dogs entering the country was assumed to be proportional to the total number of dogs in each country. Therefore, the risk of introduction of EM by dogs was assumed to be 50% and 75% of the Swedish risk for Norway and Finland, respectively. EM is present in Estonia south of Finland and infected foxes or raccoon dogs may carry the infection to Finland via the Karelian Isthmus (> 300 km) or in midwinter by passing over frozen Gulf of Finland (52-120 km). This risk is dependent on number of host-related and environmental factors difficult to assess. However, the risk was considered less than that of numerous imported dogs and was estimated on average to be 50% of the risk of dog import.

### Calculation of surveillance system sensitivity

The sensitivity was calculated annually for each surveillance system component (*SSCSe_Sp_*) and then for the whole surveillance system (*SSSe*).

#### Surveillance system component sensitivity

The annual sample sensitivity, *i.e*. the sensitivity for each sample of animals (*Sa*) within an animal species tested with test (*t*) given that the species was infected at the design prevalence for that species (P*_Sp_), was calculated as:

SasSeSp,t,y=1−[(1−Set×P*Sp)^(Ns,Sp,t,y)]

Where *Sa_s _*is the sample *s*, *Se_t _*is the sensitivity of the test *t*, *N_s, Sp, t, y _*is the number of animals in the sample *s *of species *Sp *tested with test *t *in year *y *(Table 3) and P*_Sp _is the design prevalence for the species *Sp *(Table 2).

The annual sensitivity for *SSC *for a single species and a single year, *i.e*. the probability of a positive test result in at least one individual animal in any of the samples of animals tested that year, was calculated according to the binomial distribution. For a *SSC *with two samples tested with different tests the sensitivity was calculated as:

$$SSCSe_{Sp,y}=\text{1 }–[(1-Sa_{s1}Se_{Sp,t1,y})\times (1-Sa_{s2}Se_{Sp,t2,y})]$$

Where 1- *Sa_s_Se_Sp,t,y _is *the probability of not detecting EM in the sample *s *of animals of species *Sp *tested with test *t *in year *y*.

### Calculation of the probability of freedom from EM in the country

The probability that the country is free from EM was calculated using Bayes theorem [15]. The posterior probability of freedom from infection (corresponding to the negative predictive value of a diagnostic test) was calculated for each of the 10 years as:

$$PostPFree_{y}=\text{1}-PriorPInf_{y}/(\text{1}-PriorPInf_{y}\times SSCSe_{y})\;$$

Where *PriorPInf_y _*is the pre-surveillance probability that the country is infected and *SSCSe_y _*is the sensitivity of all *SSC*s in year *y*. Although the infection has never been recorded in Sweden, Finland and Norway, a non-informative prior probability of infection (0.5) in January 2000 was used, assuming no prior information about the disease status. The *SSCSe_y _i.e*. the sensitivity of all *SSC*s in year *y*, was calculated according to the binomial distribution:

$$SSCSe_{y}=1-\prod_{Sp=1}^{5}\left(1-SSCSe_{Sp,y}\right)$$

Where 1- *SSCSe_Sp, y _*is the probability of not detecting EM in species *Sp *during year *y*.

The probability of introduction (*PIntro*) during one year *y *represents the probability that disease is introduced in the country and established at the design prevalences (P*). Either the infection may occur from a starting point of complete absence or the infection level may increase from some low level (<P*) to exceed P* during the next year, *y+*1. The prior probability that the country is infected at the beginning of *y+*1 is given by the function [15]:

$$PriorPInf_{y+1}=PostInf_{y}+PIntro_{y+1}-(PostPInf_{y}\times PIntro_{y+1})$$

Where *PriorPInf_y+1 _*is the prior probability of infection in year *y*+1, *PostInf_y _*is the posterior probability of infection in year *y *and *PIntro _y+1 _*is the probability of introduction in year *y*+1.

### Scenario analysis

A scenario analysis was performed by running two "what-if" scenarios to evaluate the effect of changes in the input variables on the probability of freedom: *i*) The prior probability of infection was decreased from 0.5 to 0.2 to include prior knowledge of absence of human cases, *ii*) The design prevalence for foxes was decreased from 1% to 0.5% and 0.05%. These estimates reflect the lowest prevalence estimate found in the literature [43,44] and 10% of the lowest reported prevalence in accordance with reasoning for the other species included in the study. Furthermore two "what-if" scenarios were run to evaluate the effect on the sensitivity of the surveillance system in the surveyed species: *i*) the sensitivity for dissection of rodents in Sweden was increased to Pert(0.8, 0.9, 0.99), *i.e*. the same as used for Finland, reflecting dissections performed by experts and *ii*) the probability of detecting EM lesions in pigs, the probability of submitting a suspected lesion to the laboratory and the probability of diagnosing a submitted sample at the laboratory was increased to Pert(0.4, 0.5, 0.6), Pert(0.8, 0.9, 0.99) and Pert(0.8, 0.9, 0.95) respectively. These values reflect what could be expected following the education of meat inspectors and assuming that all suspected lesions were tested with PCR.

### Stochastic simulation

The model was developed using Excel 2007 (Microsoft Corporation, Redmond, WA, USA) and @RISK, (Palisade, Newfield, NY, USA). The model was run with 5000 iterations for each scenario.

## Results

### Probability of freedom from EM

**Figure 3:**
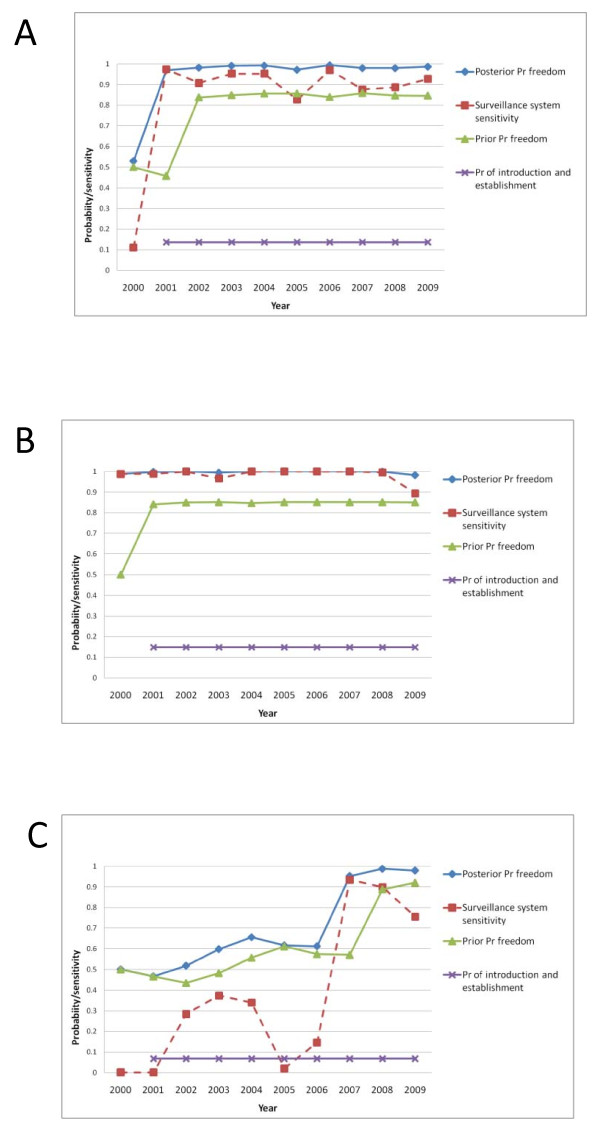
**The annual prior and posterior probability of freedom and sensitivity of surveillance systems for *Echinococcus multilocularies***. The study period is from January 2000 to December 2009. The results are presented separately per country. A = Sweden, B = Finland and C = mainland Norway.

The cumulative probability of freedom from EM in December 2009 was high in all three countries, 0.98 (95% Credibility Interval 0.96-0.99) in Finland and 0.99 (0.97-0.995) in Sweden and 0.98 (0.95-0.99) in Norway. Results from the model indicate that the probability of freedom in Finland has been high since 2000, in Sweden since 2001 and in Norway since 2007 (Figure 3).

### Surveillance system sensitivity

**Figure 4:**
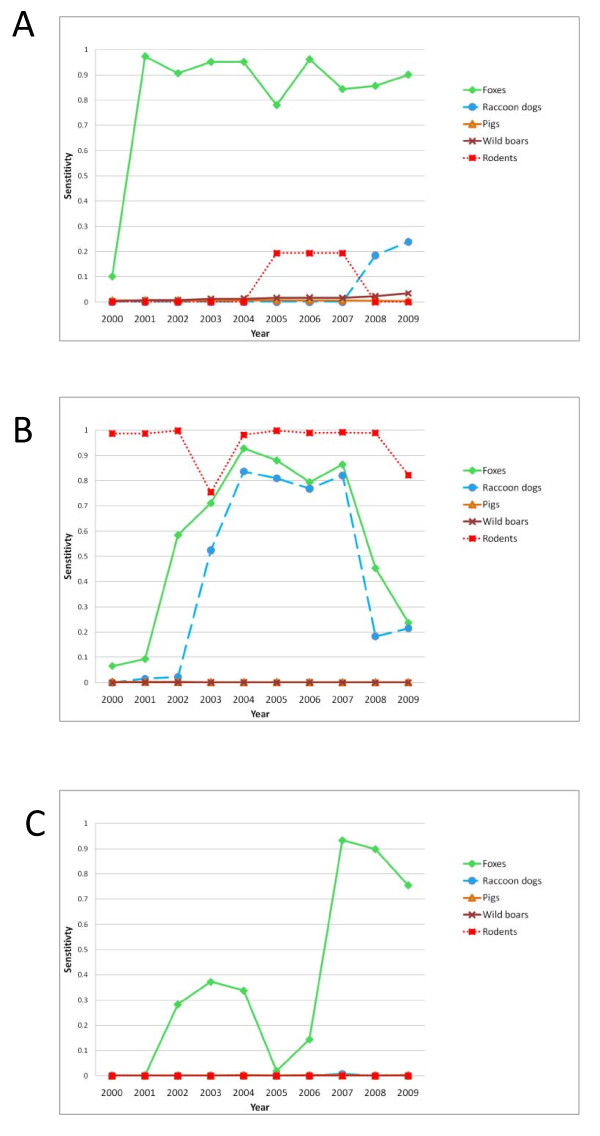
**The annual sensitivity of surveillance systems for *Echinococcus multilocularis***. The study period includes surveillance of the five different species in Sweden, Finland and mainland Norway from January 2000 to December 2009. The results are presented separately per country. A = Sweden, B = Finland and C = mainland Norway.

The estimated annual sensitivity of the surveillance system in Finland was high during the whole study period, in Sweden and Norway it was high from 2001 and 2007 respectively (Figure 3). In Finland surveillance in rodents was the component with the highest sensitivity followed by surveillance in foxes and raccoon dogs. In Sweden and Norway surveillance in foxes was the component with the highest sensitivity (Figure 4). The annual sensitivity of the surveillance system component for rodents during years 2005 to 2007 and raccoon dogs during years 2008 and 2009 in Sweden was approximately 0.2 (Figure 4A). The annual sensitivity for components domestic pigs and wild boars was below 0.01 in all countries except Sweden where the sensitivity in wild boars increased over the years from < 0.01 to 0.03 in 2009.

### Scenario analysis

**Figure 5:**
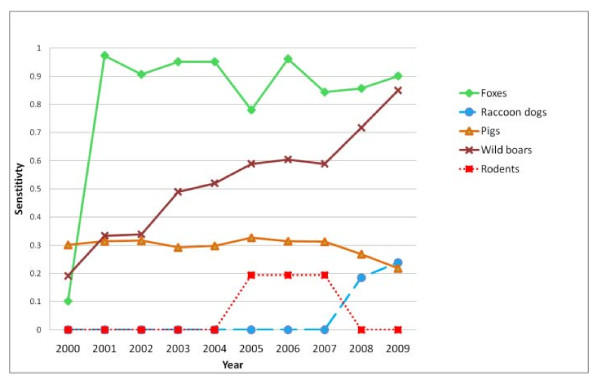
**The annual sensitivity of surveillance systems for *Echinococcus multilocularis *assuming increased sensitivity of meat inspection**. The study period is from January 2000 to December 2009 and includes surveillance of five different species in Sweden.

By reducing the prior probability of infection in the year 2000 to 0.2, the probability of freedom became slightly higher during the first years of the study period but it did not have any impact on the probability of freedom at the end of 2009. In Sweden the probability of freedom in 2000 increased from 0.53 (Figure 3A) to 0.82. In Norway, the probability of freedom between 2000 and 2006 varied between 0.47 - 0.66 (Figure 3C) and increased to 0.74 - 0.81 when using a lower prior probability of infection. For Finland and for the remaining years in Sweden and Norway, when the sensitivity of the surveillance systems as well as the probabilities of freedom were high (Figure 3), reducing the prior probability of infection did not have any major impact on the probability of freedom. When the design prevalence in foxes was decreased from 1% to 0.5% and 0.05%, the probability of freedom in 2009 decreased to 0.95 and 0.54 for Sweden and 0.90 and 0.35 for Norway while it remained at 0.98 in Finland. By increasing the sensitivity of the surveillance in rodents in Sweden to the same level as in Finland, the annual sensitivity of this surveillance system component increased from 0.20 to 0.88 in the period 2005 to 2007. Finally, increasing the overall sensitivity of meat inspection in pigs from 0.007 to 0.4 and in wild boars from 0.004 to 0.2 increased the annual sensitivity of the surveillance system component pigs and wild boars to approximately 0.3 and 0.85 in Sweden (Figure 5). In Finland and Norway it was low, < 0.2 and < 0.1 in pigs and wild boars.

## Discussion

The results of the model confirm that there is a very high probability that Sweden, Finland and mainland Norway are free from EM at the set design prevalences. Even though the surveillance differs between countries as seen in table 2, the most significant contribution to this conclusion originates from the surveillance of foxes in all three countries. However, in Finland the raccoon dog *SSC *and the vole *SSC *also had a very high sensitivity. This highlights that additional species to foxes and raccoon dogs as suggested in the EFSA report [16] can also be important in surveillance systems for EM. The sensitivity of pig *SSC *and wild boar *SSC *was low in all three countries. This is in contrast to Japan where meat inspection of pigs is considered highly informative to monitor the presence of EM [14]. However, when the sensitivity of meat inspection for pigs and wild boars was increased to 0.4 and 0.2 the annual sensitivity of these *SSC*s in Sweden increased up to approximately 0.3 and 0.8 respectively (Figure 5). This could be achieved by educating meat inspectors and laymen performing meat inspections in wild boars. Wild boars are especially interesting as they are more exposed to faeces from wild carnivores and reports from the literature show that lesions due to EM can be found in this species [21,22]. In this study this was reflected by using a higher design prevalence in wild boars compared to pigs. Therefore, surveillance in wild boars might be valuable for documenting EM status in the areas were wild boars are present, *i.e*. in the southern half of Sweden, south of latitude 61°N and to some extent also in southeastern Finland where the small wild boar population is slowly increasing. In Norway, this mode of surveillance is not yet feasible due to very low numbers of wild boars in nature. As wild boar carcasses usually are inspected by laymen, it will be crucial to document their competence in identifying EM lesions for such a surveillance strategy to get international acceptance. The results of this study are based on many assumptions. When data from literature were lacking, estimates based on expert opinion has been used. To avoid over estimate of the probability of freedom, precaucionary estimates were often used. Therefore, it was concluded that the main results of this study would still be valid, although other experts may give different estimates.

The European Food Safety Authority's recommendations for a surveillance programme to document freedom from EM [16] are based on surveillance in foxes or raccoon dogs only. However, EM has a life cycle involving several species and the first reported detection of EM in a country has been both in the main hosts such as foxes and in intermediate hosts such as voles or humans [45]. In this study, information from several species was combined into one measure for the probability of freedom of the country. Thereby, when documenting disease freedom, the number of samples per species can be adjusted so that the most cost-efficient samples are collected.

In the present study the scenario analysis showed that lowering the prior probability of infection, reflecting absence of human cases of AE, increased the probability of freedom during the first years of the study period but it did not have any impact on the probability of freedom in 2009. However if the sensitivity of the surveillance system had been lower, the effect of using a lower prior can be expected to be higher. It was concluded that absence of cases of AE, given an efficient surveillance system and compulsory notification, is important when demonstrating freedom from EM.

The choice of design prevalence is a crucial part to demonstrate freedom from infection as a "higher" level of surveillance is needed to demonstrate freedom from infection at a lower design prevalence [15]. This relation was highlighted in the scenario analysis in the present study, showing that the probability of freedom decreased when the design prevalence in foxes was decreased. When defining the target design prevalences, international standards should preferably be used [15]. However, as the design prevalences used in standards vary greatly between diseases and as no standards for EM exist, a design prevalence of 1% for foxes or raccoon dogs as suggested by EFSA, was used in the present study [16]. It could however be questioned if the same design prevalence, as suggested in the EFSA report, should be used for foxes and raccoon dogs as the prevalence in raccoon dogs are reported to be lower compared to foxes [46-48]. However, the diet of raccoon dogs is highly variable and differs geographically [49]. In Finland, the proportion of mammals in their diets appears to be higher than in Germany and Lithuania [49] and voles as food are almost equally frequent for raccoon dogs as for foxes in Finland [50]. Consequently, the same P* was used for both canid species in this study. For the remaining species, the use of 10% of the lowest prevalence estimate found in literature can be regarded as a precautionary estimate based on expert opinion.

Although surveillance of disease occurrence in different species has been included in studies to document freedom [51], to the authors' knowledge, combining results of surveillance for one disease in a number of different species using separate design prevalences has not previously been used. However, more knowledge on the expected prevalences of EM in different species is necessary to optimize the surveillance when using surveillance data from several species to document disease freedom.

In our model, the annual probability of introduction and establishment of EM was assumed to be rather high in all three countries (Figure 3) which might seem to contradict the results of the study showing that all three countries most probably are free from EM. This might be explained by EM already being introduced and established, but at a prevalence below the design prevalence. If EM was introduced the geographical spread may be very slow as reported from Japan [14]. Another possible explanation is that the risk of introduction and establishment is overestimated. The probability of introduction and establishment is based on a risk assessment for EM introduction to Sweden [41]. The true number of dogs introduced is not known and the risk assessment uses a conservative estimate that may be too high. Furthermore, the report does not differentiate between animals imported and animals in transit, the latter probably constituting in a smaller risk of excreting eggs in the environment. No data were available on the risk of establishment given introduction of an infected canid. However, the estimate used in the study may be too high as dogs travel freely between other European countries and EM is not reported to be present in all these countries. It was therefore concluded that the estimated combined risk of introduction and establishment may be too high.

According to the EFSA report, the sample size needed to document freedom from EM infection, *i.e*. to detect a prevalence of 1% with 95% confidence [16], can be collected during a five-year period, without taking into account the risk of introduction. Therefore, the surveillance systems designs assessed in this study more than fulfills the recommendations provided by the EFSA.

## Conclusions

Results indicate that there is a high probability that Sweden, Finland and mainland Norway were free from *E. multilocularis *by the end of 2009 based on surveillance results collected from January 2000 to December 2009. The study showed that the method described by Martin *et al*. could be successfully applied using surveillance data from several animal species to quantify the probability of disease freedom and to estimate the sensitivity of the surveillance systems. The scenario analyses showed that the choice of the design prevalences was important for the result and that more knowledge is needed on expected prevalences of *E. multilocularis *in infected populations of different species.

## Competing interests

MC works at the Swedish Board of Agriculture and is involved in the negotiations at the EU level concerning pet import regulations. The other authors declare that they have no competing interest.

## Authors' contributions

HW, NH, PH, MI and AW contributed to the design of the study. HW built the simulation model and performed the analysis. HW, MI, PH, GH, HU, RD and DC contributed with data to the model. All authors contributed with their expert knowledge to estimate input values to the model and also participated in writing the paper. All authors have approved the final manuscript.

## Supplementary Material

Additional file 1 **Detailed description of the data used in the study**. Click here for file
