# Supplementary material for: Combining information from surveys of several species to estimate the probability of freedom from Echinococcus multilocularis in Sweden, Finland and mainland Norway
Source: Acta Vet Scand. 2011 Feb 11;53(1):9. doi: 10.1186/1751-0147-53-9 (PMC3049754; doi:10.1186/1751-0147-53-9)
Supplement: Additional file 1 — Detailed description of the data used in the study. [file 1751-0147-53-9-S1.DOC]

# Supplementary material 1:

# Combining information from surveys of several species to estimate the probability of freedom from *Echinococcus multilocularis* in Sweden, Finland and mainland Norway

Helene Wahlström^1§^, Marja Isomursu^2^, Gunilla Hallgren^1^, Dan Christensson^1^, Maria Cedersmyg^3^, Anders Wallensten^4^, Marika Hjertqvist^4^, Rebecca Davidson^5^, Henrik Uhlhorn^1^, Petter Hopp^5^

^1^National Veterinary Institute, 752 89, Uppsala, Sweden

^2^Finnish Food Safety Authority Evira, Fish and Wildlife Health Research Unit, PL 517, 90101 Oulu, Finland

^3^Swedish Board of Agriculture, 551 82 Jönköping, Sweden,

^4^Swedish Institute for Communicable Infectious Disease Control, 171 82 Stockholm, Sweden

^5^Norwegian Veterinary Institute, 0106 Oslo, Norway

# Data sources

## Sweden

### Red foxes

Data on the number of examined foxes and raccoon dogs were obtained from the National Veterinary Institute (SVA). Fox carcasses were obtained from hunters from all over Sweden. Faecal samples were analysed with a coproantigen ELISA (CoA) [1] at the Institute of Parasitology, Zürich University. In positive animals, the intestines were examined with sedimentation and counting technique (SCT) [2] at SVA. In some cases foxes were examined with SCT without previous testing with CoA (Table 2).

### Raccoon dogs

In a project conducted during 2008 and 2009, intestines from 49 raccoon dog carcasses were examined with SCT (Table 2). The project was run by SVA, the Swedish Association for Hunting and Wildlife Management, Swedish University of Agricultural Sciences, the Swedish Environmental Protection Agency and the County Administrative Boards in the counties of Norrbotten and Västerbotten. All raccoon dogs were killed by shooting. The raccoon dogs originated from the northern part of Sweden as raccoon dogs do not occur in the southern parts of the country.

## Domestic pigs

Pigs not intended for private consumption have to be submitted for meat inspection and samples of organs with suspected lesions can be sent to SVA for further investigation without cost. The number of meat-inspected fattening pigs and sows were obtained from the National Board of Agriculture. Information needed to estimate the proportion of pigs that have had access to pasture was obtained from the Swedish Animal Health Service (SvDHV). EU requirements for organic pig farming implemented in 2008 only require outdoor visits, but Swedish organic animal husbandry requires pigs to be on pasture at least four months during the summer. The annual number of slaughtered organic pigs was obtained from the SvDHV. Approximately 25% (2008) and 75% (2009) of organic pigs were estimated to be raised according to EU regulations and therefore excluded from the analysis. Furthermore, as only pigs raised during the summer had access to pasture, only 50% of pigs raised according to Swedish organic animal husbandry were included in the study. As only data on the annual number of slaughtered sows was available, the number of slaughtered sows assumed to have been on pasture was estimated as: *a* (*b*/*c*), where *a*=annual number of slaughtered sows, *b*=number of slaughtered fattening pigs included in the study and *c*=total number of fattening pigs slaughtered (Table 2).

## Free-ranging wild boars

The number of free ranging wild boars hunted annually was obtained from the national game bag statistics by the Swedish Association for Hunting and Wildlife Management. According to the national legislation, wild animals susceptible to trichinosis should always be delivered to a game-handling establishment approved by the National Food Administration according to regulation (EC) No 853/2004 except when the meat is intended for private domestic consumption. Since 1 January 2006, a hunter or a trained person as described in regulation (EC) No 853/2004 can after examination of the wild game deliver the carcass to the game-handling establishment for further examination. In the present study, all wild boars shot were assumed to be meat inspected, as those intended for private consumption will be inspected by the hunter. Any suspected lesions can be sent to SVA for further investigation without cost. Only free-ranging wild boars were included in the study. As the liver is assumed to be damaged by the shot in approximately 5% of cases (pers. com. Andersson, PO.) only 95% of wild boars shot were included in the study (Table 2).

## Rodents

Data on the number of examined voles was obtained from the Swedish Institute for Infectious Disease Control. Dissection of rodents was performed by laypersons within projects not related to EM (Table 2).

# Norway

## Red foxes

Faecal samples were collected from red foxes shot during the licensed hunting seasons between July and April. During 2002-2005, hunters were contacted by sending invitation letters to those who had supplied pelts to the Oslo Fur Auction House. For the 2006 to 2009 hunting seasons, hunters were invited based on the list of registered fox hunters (Statistics Norway). Fox hunted from all counties in Norway were represented. Faecal samples collected between 2002 and 2005 were examined at the University of Zürich using CoA [3]. All positive samples were examined with a modified taeniid egg isolation [4, 5] and multiplex PCR [6]. Material collected from 2006 to 2009 was examined with a modified taeniid egg isolation and multiplex PCR as described by Davidson [7].

## Raccoon dogs

The raccoon dog is considered an unwanted species in Norwegian fauna. Raccoon dogs shot should be submitted to the National Veterinary Institute and will be routinely examined for several infections including EM.

## Pigs

Pigs not intended for private consumption have to be submitted for routine meat inspection and lesions suspected of echinococcosis or other notifiable diseases can be sent to National Veterinary Institute for further investigation without cost.

It is a requirement for organic pig farming to have access to outdoor visits and approximately 75% of these had access to pasture. When there was access to pasture, all sows and 50% of the fattening pigs were considered to have had access to pasture during their lifetime. The annual numbers of fattening pigs slaughtered and sows originating from the organic pig herds were obtained from the Norwegian Agricultural Authority for the years 2003 to 2009. For 2000-2002 data on organic pig farming had not been collected and the number of animals was estimated to be 2/3 of 2003 data.

## Wild boars and rodents

The number of wild boars in Norway is considered to be less than 500 and there are no registered meat inspections for this species. We are not aware of any examinations of rodents for EM in Norway.

# Finland

## Foxes and raccoon dogs

Foxes and raccoon dogs were collected for the national surveillance programme of rabies, trichinellosis and echinococcosis and examined at the Finnish Food Safety Authority (Evira). Most animals were hunted during the hunting season; some were found dead. Foxes were obtained from all over the country with emphasis on southeastern and northernmost Finland. Raccoon dogs occur abundantly only in the southern half of Finland and samples were obtained mostly from southeastern Finland. In 2000-2007, faecal samples were examined by Chekit ELISA (Bommeli Diagnostics, Bern, Switzerland) and positive animals were analysed by SCT. In 2008-2009 faecal samples were analysed with a CoA at the Institute of Parasitology, Zürich University, and faecal samples from positive animals were examined by egg isolation (faecal flotation) at Evira, and, if eggs were found, subsequent egg PCR at Zürich University.

## Pigs

The number of meat-inspected fattening pigs and sows and the estimate of the proportion of pigs in organic production were obtained from Evira, Control Department. Finnish pigs in organic farms are required to have access to pasture or corrals with possibilities for natural behaviour for a minimum of six months (May-October) per year.

Meat inspection is mandatory for pigs intended for commercial use. Samples of any suspicious organ lesions found at meat inspection can be sent to Evira, Research Department, for further investigation without cost.

## Wild boars

Free-ranging wild boars are relatively rare in Finnish nature and occur mostly in southeastern Finland, close to the Russian border. The annual hunting bag is small and hunted wild boars are usually consumed privately without official meat inspection. In this study, only farmed, meat-inspected wild boars were included in the analysis. Farmed wild boars are raised in outdoor pastures. The number of animals was obtained from Evira.

## Rodents

Small rodents, mainly voles, are trapped regularly in designated areas as well as sporadically in various places all over Finland by the Finnish Forest Research Institute (Metla) in order to monitor local population changes. Voles are dissected by experienced biologists specializing in small mammals with knowledge of the morphology of liver parasites in voles. The number of voles investigated was estimated by Metla.

# Surveillance in humans

Surveillance for echinococcosis in humans in Sweden, Finland and Norway is based on passive surveillance. As patients eventually will seek medical care, this is considered sufficient for detecting clinical cases of alveolar echinococcosis AE (*E. multilocularis*) and also cystic echinococcosis CE (*E. granulosus*). Echinococcosis has been notifiable for both clinicians and laboratories since 2004, 1994 and 2003 for Sweden, Finland and Norway, respectively. In the earlier part of the study period, surveillance relied on voluntary reporting by the laboratories. CE is not reported separately from AE in the surveillance system; however, discrimination between the two forms of the disease has been performed at the laboratory. *E. multilocularis* has never been reported in humans in Sweden, Finland and Norway. Since echinococcosis became notifiable up to and including 2009, Sweden, Finland and Norway have reported 79, 12 and 15 human cases of CE, respectively. All cases have been diagnosed in immigrants from countries where the disease is considered endemic and are therefore considered as being imported.

# References

1. Deplazes P, Alther P, Tanner I, Thompson RC, Eckert J: ***Echinococcus multilocularis* coproantigen detection by enzyme-linked immunosorbent assay in fox, dog, and cat populations.** *J Parasitol* 1999, **85:**115-121.

2. Eckert J, Gottstein B, Heath D, Liu F-J: **Prevention of echinococcosis in humans and safety precautions.** In *WHO/OIE Manual on Echinococcosis in Humans and Animals: a Public Health Problem of Global Concern.* Edited by Eckert J, Gemmell MA, Meslin F-X, Pawłowski ZS. Paris: World Organisation for Animal Health; 2001: 238-247

3. Deplazes P, Eckert J: **Diagnosis of the *Echinococcus multilocularis* infection in final hosts.** *Appl Parasitol* 1996, **37:**245-252.

4. Stefanic S, Shaikenov BS, Deplazes P, Dinkel A, Torgerson PR, Mathis A: **Polymerase chain reaction for detection of patent infections of *Echinococcus granulosus* ("sheep strain") in naturally infected dogs.** *Parasitol Res* 2004, **92:**347-351.

5. Mathis A, Deplazes P, Eckert J: **An improved test system for PCR-based specific detection of *Echinococcus multilocularis* eggs.** *J Helminthol* 1996, **70:**219-222.

6. Trachsel D, Deplazes P, Mathis A: **Identification of taeniid eggs in the faeces from carnivores based on multiplex PCR using targets in mitochondrial DNA.** *Parasitology* 2007, **134:**911-920.

7. Davidson RK, Øines Ø, Madslien K, Mathis A: ***Echinococcus multilocularis* – adaptation of a worm egg isolation procedure coupled with a multiplex PCR assay to carry out large scale screening of red foxes (*Vulpes vulpes*) in Norway.** *Parasitol Res* 2009, **104***:* 509-514.
